# Notes and News

**Published:** 1905-02-25

**Authors:** 


### NOTES AND NEWS.

The ultimate cost of the New Royal Infirmary, Manchester, is estimated as likely to reach half a million.

It is interesting to learn that Professor von Hausemann reports that a *post mortem* on the celebrated painter Adolf von Menzel reveals the fact that he was hydrocephalic.

An interesting feature of the automobile show at Olympia, from our point of view, was the number of exhibits which had been sold to medical men. They represented the simplest form of small car to the finest electric brougham for the wealthy consultant.

The total number of cases of typhoid fever at Lincoln up to Tuesday night was 622, and the deaths 60. The Corporation have decided to obtain a fresh supply of water for the city, and meanwhile water is being carted from ten miles away, and carts are located at different places for people to help themselves.

The Prussian railways have been provided, on their express routes, with railway carriages transformable into ambulance compartments for the use of sick passengers. Some time ago we pointed out in these columns that our southern lines especially should be provided with suitable accommodation for invalids. Were accommodation available at moderate cost, the lines to seaside resorts would certainly benefit financially.

We have received a circular from the London Association for the Prevention of Premature Burial. Under the objects of the association we read "The safeguarding of the members of the association against premature burial by means of an arrangement with medical experts who have devoted special attention to death verification," and then follows a list of names of medical "experts" in various towns who we presume are prepared to come in and verify or refute the verdict passed by the medical attendant of any member.

A new small-pox hospital has been erected at Townhill for Dunfermline. It is to contain 20 patients.

The late Mr. A. G. Hubbuck, of Rotherfield, has bequeathed £2,000 to the East London Hospital for Children and £1,000 to the Chelsea Hospital for Women. The late Mrs. M. Worthington, of Chester, has bequeathed £1,000 to the Salford Sick-poor Nursing Institute.

Through the generosity of Mrs. W. H. Martin, a pavilion for light and electric treatment has been opened at the Cardiff Infirmary. The building comprises an *x*-ray room, high-frequency sinusoidal room, Finsen lamp room, photographic and dark rooms, and a dressing and waiting room.

According to Lieut.-Col. Valentine Matthews the Volunteer Royal Army Medical Corps is suffering through the compulsory camp clauses. In 1904 the London corps numbered the lowest, and the difficulty of obtaining fresh volunteers is very great. He expressed a wish that the Government would abolish the compulsory camp clauses in their case for a year or two.

A paper on "The Control of Hospital Expenditure with Efficiency" will be read by the medical superintendent of the Glasgow Western Infirmary, Dr. Donald Mackintosh, before the Hospitals Association at Charing Cross Hospital on Friday, March 3rd, at 8 o'clock. Tickets can be obtained from the Hon. Secretary, the Hospital Building, 28 Southampton Street, Strand, W.C.

The Drapers' Company has promised to contribute £10,000 to the King's College Hospital Removal Fund if it has reached £300,000 by December 31st, 1905. They previously promised £5,000 if £300,000 was forthcoming by December 31st, 1904. Already £120,000 has been collected, and it is to be hoped that the generous contributions made by the King's Fund and the promise held out by the Drapers Company will stimulate a large number of givers before the end of the year.

A conference is to be held on Thursday, March 2nd, at 20, Hanover Square, at 4 p.m., for the purpose of considering the formation of a Convalescent Homes Association. The object of the association will be to promote co-operation amongst existing convalescent institutions with a view to increasing their power of supplementing the work of the hospitals, exchanging administrative experience and promoting economy. It will be a great advantage if medical men are able to select an institution especially suited to the requirements of individual cases, instead of sending all classes of patients to one because it only happens to be available. If a combination can be effected amongst the convalescent homes it must be beneficial to all. The Presidents of the Royal Colleges of Physicians and Surgeons, and many representatives of medical institutions, the medical profession, and of a number of convalescent institutions, have promised to attend, and the meeting will be both interesting and important.

